# The association between acupuncture and response to immune checkpoint inhibitors in non-small cell lung cancer

**DOI:** 10.1186/s13020-025-01148-4

**Published:** 2025-09-19

**Authors:** Rui Zhou, Yan-juan Zhu, Yi-han He, Jia-jia Lin, Ze-xin Zhang, Wen-jie Zhao, Hao-chuan Ma, Xue-song Chang, Ya-dong Chen, Wen-zhu Li, Xian Chen, Xiao-shu Chai, Hai-bo Zhang

**Affiliations:** 1 Department of Oncology, The Second Affiliated Hospital of Guangzhou University of Chinese Medicine, Guangdong Provincial Hospital of Traditional Chinese Medicine, No. 111, Dade Road, Guangzhou, 510120 Guangdong China; 2 Guangdong-Hong Kong-Macau Joint Lab on Chinese Medicine and Immune Disease Research, Guangzhou, China; 3 Guangdong Provincial Key Laboratory of Clinical Research on Traditional Chinese Medicine Syndrome, Guangzhou, China; 4 Guangzhou University of Chinese Medicine, Guangzhou, China; 5 State Key Laboratory of Dampness, Syndrome of Chinese Medicine, The Second Affiliated Hospital of Guangzhou University of Chinese Medicine, Guangzhou, China

**Keywords:** Acupuncture, Immune checkpoint inhibitors, Non-small cell lung cancer, Immunotherapy

## Abstract

**Background:**

Immune checkpoint inhibitors (ICIs) have transformed non-small cell lung cancer (NSCLC) treatment, yet low response rates and resistance remain challenges. Emerging evidence suggests acupuncture may enhance ICI effectiveness by reprogramming immunosuppressive tumor microenvironments.

**Methods:**

A retrospective analysis was conducted on 217 ICI-treated NSCLC patients. Patients who initiated acupuncture before median progression-free survival (mPFS, 8.87 months) of the overall population were classified as the acupuncture group (n = 97). Cox regression analysis was employed to evaluate the association between clinical characteristics and outcomes. Propensity score matching (PSM) was performed to mitigate selection bias. Subgroup and interaction analyses were conducted to evaluate potential effect modifiers.

**Results:**

Acupuncture was significantly associated with improved mPFS (10.23 vs. 7.87 months, *P* = 0.036), while a numerical trend favoring acupuncture was observed in overall survival (mOS: 24.1 vs. 20.9 months, *P* = 0.352). Obvious benefits emerged in bone metastasis patients, with the acupuncture group reducing progression risk by 76% (HR = 0.24, 95% CI 0.14–0.42, *P* < 0.001) and death risk by 53% (HR = 0.47, 95% CI 0.21–1.03, *P* = 0.049). Conversely, PD-L1-positive subgroups exhibited no trend of improvement (subgroup HR > 1.0), with interaction HRs > 2.0 indicating potential impairment of ICI efficacy. The results of post-PSM data suggest that acupuncture was independently associated with improved PFS (aHR = 0.62, 95% CI 0.41–0.94, *P* = 0.024).

**Conclusion:**

Our findings suggest a positive association between acupuncture and improved outcomes in ICI-treated NSCLC patients, particularly in those with bone metastasis, underscoring the potential role of acupuncture in the integrative management of cancer. However, biomarker-guided patient stratification is essential for integrating acupuncture into oncology care.

**Supplementary Information:**

The online version contains supplementary material available at 10.1186/s13020-025-01148-4.

## Introduction

Lung cancer is one of the most lethal diseases globally [1], posing a major public health challenge that demands urgent resolution. While immune checkpoint inhibitors (ICIs) have improved outcomes for patients with driver-negative non-small cell lung cancer (NSCLC) [2], clinical limitations persist: only approximately 23.2% of treatment-naive patients derive significant benefit from ICIs, with the majority developing primary or secondary resistance [3]. This low response rate critically restricts the efficacy of ICIs, underscoring the imperative to identify strategies for enhancing ICI efficacy and improving NSCLC patients'long-term survival.

ICI resistance mechanisms arise from multidimensional interactions among tumor cells, the immune system, and the tumor microenvironment (TME) [4]. First, Tumor cells may evade immune recognition through genetic or epigenetic changes that reduce immunogenicity, and immune editing promotes the accumulation of low-immunogenic clones, which ultimately drive resistance [5]. Second, the TME establishes an immunosuppressive network through recruitment of regulatory T cells (Tregs) [6], myeloid-derived suppressor cells (MDSCs) [7, 8], and other inhibitory immune cells. In addition to affecting T cell function, these components also create metabolic competition and physical barriers that impair effector T cell infiltration and function [9, 10]. Furthermore, chronic tumor antigen exposure induces T cell exhaustion, characterized by elevated expression of inhibitory receptors (for example, TIM-3 and LAG-3), which further diminishes ICIs effectiveness [11].

Notably, TME heterogeneity directly dictates ICI responsiveness [12]."Hot tumors"—characterized by abundant CD8^+^ T cell infiltration and tertiary lymphoid structure formation—exhibit heightened ICI sensitivity. In contrast,"cold tumors"demonstrate marked resistance, displaying either immune exclusion (with immune cells confined to tumor margins) or desertification (lacking immune infiltrates), coupled with suppressive cell dominance and dense stromal barriers. Current therapeutic strategies focus on TME reshaping [13], particularly through converting “cold” tumors to “hot” phenotypes by combination approaches such as chemotherapy or anti-angiogenic agents—a paradigm increasingly adopted in clinical practice.

Acupuncture has gained recognition as an integrative oncology intervention [14], with demonstrated effectiveness in alleviating cancer-related symptoms and mitigating treatment-induced toxicities [15–17]. Emerging clinical evidence suggests broader immunomodulatory effects, including upregulation of proinflammatory cytokines (including IL-2, IL-12, TNF-α, and IFN-γ) and downregulation of anti-inflammatory mediators (including IL-4, IL-6, and IL-10). Intriguingly, studies in tumor-bearing animal models indicated that acupuncture may activate the tumor immune microenvironment (TIME) and potentiate anti-tumor immunity [18, 19], raising the possibility of converting “cold” tumors to “hot” states.

Despite these mechanistic insights, the clinical effectiveness of acupuncture as an ICI adjuvant in NSCLC remains unexplored. This retrospective study aims to address this knowledge gap by evaluating whether acupuncture is associated with improved survival outcomes in NSCLC patients receiving ICI therapy.

## Materials and methods

This study was approved by ethics committee of our hospital (Ethics Committee of Guangdong Provincial Hospital of Traditional Chinese Medicine, ZE2021-241), with waiver of written informed consent for this study. All patients have provided their written informed consent before anti-tumor treatments.

### Patient selection

We conducted a retrospective analysis of patients with locally advanced or metastatic NSCLC who were admitted to the Oncology Department of Guangdong Provincial Hospital of Traditional Chinese Medicine between January 2018 and December 2021. Patients were eligible for inclusion if they had been diagnosed pathologically with NSCLC and had received ICI-based regimens as first-line treatment. The exclusion criteria included patients who received ICIs as perioperative treatments rather than systemic therapy, those harboring sensitive driver gene mutations treated with targeted therapy, those with other uncontrolled malignancies, and those who refused follow-up.

Clinical characteristics and treatment experiences were collected from the electronic medical record system. All patients were followed up until September 2023 or until death. Based on the 10 events per variable rule [20, 21], this sample size with approximately 80% events was sufficient for the multivariate regression analyses.

### Definition of acupuncture

Longer survival durations in NSCLC patients may inherently increase exposure to acupuncture due to more frequent hospitalizations, potentially introducing immortal time bias. To address this confounding factor, we rigorously defined the acupuncture group as patients who initiated acupuncture therapy before the median PFS (mPFS) of the overall cohort (8.87 months). The acupuncture treatments included manual acupuncture, electroacupuncture, auricular acupuncture, and intradermal acupuncture.

### Covariates

The clinical information at the time of initial ICI administration was used as the baseline data. The baseline information included age, gender, smoking history, pathology, clinical stage, and line of treatment. In addition, a variety of factors that have previously been reported as potential biomarkers of the efficacy of ICI were also collected as potential covariates, including PD-L1 expression, genomic status, eastern cooperative oncology group performance status (ECOG-PS), and combination therapy.

### Outcomes

The outcomes included PFS and OS, which were assessed using the Response Evaluation Criteria in Solid Tumors (RECIST, version 1.1). PFS was defined as the period from the initiation of ICI to tumor progression, as determined by imaging or death from any cause. OS was defined as the time from the first ICI treatment to death from any cause.

### Statistical analysis

The retrospective data were recorded independently in EpiData (version 3.1) by two authors, and reviewed by another author. Categorical data were reported as counts and percentages. Comparisons between ratios were performed using the Chi-square test or Fisher exact test where appropriate. Missing values for covariates were imputed using the multiple imputation by chained equations (MICE) method with 5 iterations. Five datasets were generated, and the pooled result of 5 imputed data was used. Propensity score matching (PSM) was performed to reduce potential biases due to imbalances of baseline data between the acupuncture group and control group.

The effect of acupuncture on ICI-treated NSCLC patients was evaluated using a Cox proportional hazard regression model. Individuals who did not receive acupuncture treatment during ICI therapy or receive acupuncture after the mPFS of the overall population were assigned to the control group. Covariates associated with PFS or OS (*P* ≤ 0.2) were included in the adjusted Cox regression model. The results are presented as hazard ratios (HRs) and adjusted-HRs (aHRs) with 95% confidence intervals (CIs). Survival analysis was performed using the Kaplan–Meier method and the Log-rank test. A two-sided *P* value less than 0.05 was considered statistically significant.

Pre-designed subgroup analysis was performed to identify influencing factors for acupuncture combined with ICIs-based therapy. Clinical characteristics were taken into consideration, including smoking history, PS score, pathology, clinical stage, PD-L1 expression, bone metastasis, brain metastasis, liver metastasis, epidermal growth factor receptor (*EGFR*) mutation, PD-L1 expression, the types of ICI. The Cox regression model was also employed to analyze the interaction between acupuncture and other characteristics. All analyses were carried out using R software (R Statistical Software, version 4.1.3) with the R packages “tidyverse”, “mice”, “VIM”, and “survival”.

## Result

### Population inclusion and follow-up

A total of 466 NSCLC patients treated with ICIs-based regimens were reviewed. Six patients received ICIs as neo-adjuvant treatment, and 48 patients declined follow-up. Of the remaining patients, 99 received ICIs as second-line treatment, and 91 as third-line or later treatment. Five NSCLC patients harboring *EGFR*-sensitizing mutations were excluded from the analysis due to protocol deviation: they received only one cycle of ICIs-based therapy prior to confirmation of their *EGFR* mutation status, and subsequently transitioned to *EGFR*-tyrosine kinase inhibitor treatment. Finally, 217 patients were included (Table 1), of which 97 (44.7%) were assigned to the acupuncture group and 120 (55.3%) to the control group. Until September 2023, 176 (81.1%) patients had progressed, and 133 (61.3%) patients had died. The mPFS for the total population was 8.87 months (95% CI 7.3–10.8 months), and the median OS (mOS) was 21.6 months (95% CI 17.9–29.3 months).

**Table 1:** Demographic and clinical characteristics of the 217 patients with NSCLC treated with immune checkpoint inhibitors-based therapy

Clinical characteristics	Overall population	Acupuncture group	Control group	P value
	217	97 (44.7)	120 (55.3)	
Age (mean ± SD)	63.30 ± 9.05	63.80 ± 9.32	62.90 ± 8.83	0.468
Gender (%)				0.341
Male	181 (83.4)	84 (86.6)	97 (80.8)	
Female	36 (16.6)	13 (13.4)	23 (19.2)	
Smoke history (%)				0.384
Never smoke	52 (24.2)	20 (20.8)	32 (26.9)	
Smoke	163 (75.8)	76 (79.2)	87 (73.1)	
Missing	2	1	1	
Pathology (%)				0.361
Adenocarcinoma	130 (59.9)	62 (63.9)	68 (56.7)	
Squamous cell carcinoma	68 (31.3)	29 (29.9)	39 (32.5)	
Others	19 (8.8)	6 (6.2)	13 (10.8)	
ECOG PS (%)				0.808
0	40 (20.7)	17 (18.1)	23 (23.2)	
1	113 (58.6)	59 (62.8)	54 (54.6)	
2	30 (15.5)	13 (13.8)	17 (17.2)	
3	9 (4.7)	5 (5.3)	4 (4.0)	
4	1 (0.5)	0	1 (1.0)	
Missing	24	3	21	
T stage (%)				0.107^a^
T0	7 (3.6)	1 (1.1)	6 (5.8)	
T1	15 (7.7)	7 (7.7)	8 (7.8)	
T2	29 (15.0)	17 (18.7)	12 (11.7)	
T3	31 (16.0)	10 (11.0)	21 (20.4)	
T4	112 (57.7)	56 (61.5)	56 (54.4)	
Missing	23	6	17	
N stage (%)				0.923
N0	24 (12.2)	12 (13.2)	12 (11.3)	
N1	2 (1.0)	1 (1.1)	1 (0.9)	
N2	80 (40.6)	35 (38.5)	45 (42.5)	
N3	91 (46.2)	43 (47.3)	48 (45.3)	
Missing	20	6	14	
M stage (%)				0.480^a^
M0	55 (27.4)	29 (30.2)	26 (24.5)	
M1	146 (72.6)	67 (69.8)	79 (75.2)	
Missing	16	1	15	
Clinical stage (%)				0.645^a^
IIIA	16 (7.8)	10 (10.4)	6 (5.6)	
IIIB	30 (14.7)	14 (14.6)	16 (14.8)	
IIIC	12 (5.9)	5 (5.2)	7 (6.5)	
IV	146 (71.6)	67 (69.8)	79 (73.2)	
Missing	13	1	12	
The number of metastatic organ (%)				0.835
0	16 (9.9)	6 (8.2)	10 (11.4)	
1	59 (36.6)	29 (39.7)	30 (34.1)	
2	35 (21.7)	18 (24.7)	17 (19.3)	
3	24 (14.9)	9 (12.3)	15 (17.0)	
4	15 (9.3)	6 (8.2)	9 (10.2)	
≥ 5	12 (7.5)	5 (6.8)	7 (8.0)	
Missing	56	24	32	
Liver metastasis (%)				0.104
No	131 (84.5)	65 (90.3)	66 (79.5)	
Yes	24 (15.5)	7 (9.7)	17 (20.5)	
Missing	62	25	37	
Bone metastasis (%)				0.686
No	75 (50.3)	33 (47.8)	42 (52.5)	
Yes	74 (49.7)	36 (52.2)	38 (47.5)	
Missing	68	28	40	
Brain metastasis (%)				0.510
No	115 (77.7)	55 (80.9)	60 (75.0)	
Yes	33 (22.3)	13 (19.1)	20 (25.0)	
Missing	69	29	40	
PD-L1 (%)				0.234
< 1%	23 (18.7)	6 (11.5)	17 (23.9)	
1–49%	48 (39.0)	24 (46.2)	24 (33.8)	
≥ 50%	52 (42.3)	22 (42.3)	30 (42.3)	
Missing	94	45	49	
The types of ICI (%)				0.918
PD-1	207 (95.4)	93 (95.9)	114 (95.0)	
PD-L1	3 (1.4)	1 (1.0)	2 (1.7)	
Others	7 (3.2)	3 (3.1)	4 (3.3)	
Chemotherapy (%)				0.058
No	50 (23.0)	16 (16.5)	34 (28.3)	
Yes	167 (77.0)	81 (83.5)	86 (71.7)	
Targeted therapy (%)				0.502
No	145 (66.8)	62 (63.9)	83 (69.2)	
Yes	72 (33.2)	35 (36.1)	37 (30.8)	

*ECOG PS* Eastern cooperative oncology group performance status, *PD-L1* Programmed cell death1 ligand1

^a^Fisher’s exact test

### Clinical characteristics of the included population

The average age of the study cohort was 63.3 ± 9.05 years, with 36 females (16.6%) and 163 patients (78.4%) having a history of smoking. Among the patients, 130 (59.9%) were diagnosed with adenocarcinoma, 68 (31.3%) with squamous cell carcinoma. There were no statistically significant differences between the acupuncture and control groups regarding age (*P* = 0.468), gender (*P* = 0.341), smoking history (*P* = 0.384), or pathological type (*P* = 0.361).

No significant differences in PS scores were observed between the two groups (*P* = 0.808). However, the control group had more missing values. We found that the PS scores and clinical stage of 11 patients were unavailable because they received their first-line treatment at other hospitals. No significant differences in clinical stage were found between the groups. The acupuncture group had a higher proportion of T4 and T2 stages, while the control group had more T0, T1, and T3 stages (T, *P* = 0.107; N, *P* = 0.923; M, *P* = 0.480; Clinical stage, *P* = 0.645). There were no significant differences between the groups in terms of metastatic lesions (number of metastatic organs, *P* = 0.835; Liver metastasis, *P* = 0.104; Bone metastasis, *P* = 0.686; Brain metastasis, *P* = 0.510).

### Molecular characteristics

In the current study, four patients with *EGFR* mutation were included, all of whom were in the control group. Three of the four patients had *EGFR exon 20 insertion*, and one had the *EGFR exon 21 c.2281G* > *T* mutation. Among the 85 patients with available *KRAS* status, 45 had *KRAS* mutation, with 23 in the acupuncture group and 22 in the control group. The acupuncture group included 2 patients with *BRAF* mutation, 3 with *ERBB2* mutation, 2 with *MET* mutation, and 1 with a *PIK3CA* mutation. The control group included 2 patients with *BRAF* mutation, 2 with *ERBB2* mutation, and 4 with *PIK3CA* mutation. There were no patients with known *ALK* (0/123) or *ROS1* (0/116) mutations in the overall population.

Among 217 patients, PD-L1 expression level were available for 123 (56.7%). Of these, 52 patients (42.3%) exhibited strongly positive PD-L1 expression (defined as TPS ≥ 50% or CPS ≥ 10), while 23 (18.7%) had intermediate positivity (TPS 1–49% or CPS 1–9). In the acupuncture group, 6 patients had PD-L1-negative tumors compared to 17 in the control group, with no significant between-group difference in PD-L1-negative distribution (*P* = 0.234). Among strongly positive PD-L1 patients, the acupuncture group included 22 cases versus 30 in the control group.

### Treatment information of the included population

The median number of ICI sessions was 6 (IQR: 3–12 sessions), and the median number of acupuncture sessions was 5 (IQR: 2–10 cycles). Among the overall population, 95.4% received PD-1 inhibitors, 77.0% combined with chemotherapy, 33.2% underwent targeted therapy, 6.5% in combination with radiotherapy, and 1.8% with pulmonary ablation therapy in the first-line treatment (Table 1).

Among the 166 patients who received chemotherapy, 126 patients (75.9%) accepted double-drug chemotherapy, 35 (21.1%) received single-agent chemotherapy, and 5 had other regimens. Among the 5 patients, one in the control group switched from the GP regimen to the TP regimen due to thrombocytopenia. One patient, in the acupuncture group, was treated with oral chemotherapy (vinorelbine), while the remaining three switched treatments due to intolerance. A higher proportion of patients in the acupuncture group received chemotherapy compared to controls (83.5% vs. 71.7%, *P* = 0.058).

Seventy-two patients received targeted therapy in addition to ICIs, with the majority combining it with bevacizumab (49/72) or anlotinib (19/72). The five patients who received other targeted regimens included two with apatinib, one with osimertinib (*EGFR exon 20 insertion*), one with endostar, and one with the combination of bevacizumab intrapleural infusion and oral anlotinib.

Among the patients who received pulmonary ablation therapy, there was 1 in the acupuncture group and 3 in the control group. One patient underwent ablation due to oligoprogression of a pulmonary lesion in the control group, while the remaining patients received ablation for palliative tumor reduction. Fourteen patients received radiotherapy, with 6 in the acupuncture group and 8 in the control group.

### Association of characteristics with clinical outcomes

The mPFS of smokers and non-smokers was similar (8.77 vs. 9.17 months, Figure S1A), but smokers showed a poorer mOS (20.5 vs. 25.6 months, Figure S1B). Compared to squamous cell carcinoma, adenocarcinoma patients had a better prognosis (mPFS, 9.33 vs. 7.63 months; mOS, 21.57 vs. 16.80 months, Figure S2). Patients with a PS score of 1 and 2 had an mPFS of 10.27 and 6.45 months, and an mOS of 27.03 and 10.50 months, respectively, while those with missing values had an mPFS of 7.6 months and mOS of 20.9 months (Figure S3). In our cohort, stage III patients had better outcomes than stage IV patients (IIIA vs. IIIB vs. IIIC vs. IV, mPFS: 21.67 vs. 9.97 vs. 22.60 vs. 7.87 months; mOS: not reached *vs.* 36.8 vs. 40.1 vs. 18.5 months, Figure S4). The number of metastatic organs reflects tumor burden, with an increased number of metastatic organs or the presence of liver, bone, or brain metastasis associated with a poorer prognosis (Figure S5-S8).

In the overall population, the PFS of the 4 patients with non-sensitive *EGFR* mutations was similar to that of other groups, but they were likely to show a better OS (mOS, 30.0 months). NSCLC patients with *KRAS* mutation had longer PFS than those with wild-type *KRAS* mutation (mPFS 13.7, 95% CI 6.77–20.6), though no OS benefit was observed. The PFS of patients with mutant *BRAF* in the acupuncture group were 5.2 and 20.3 months, while in the control group, the PFS were 18.4 and 37.3 months. Five patients with *ERBB2* mutations were included, all showing shorter PFS, with 3 in the acupuncture group and 2 in the control group. Two patients with *MET* mutation were included in the acupuncture group, with PFS of 5.17 and 9.77 months. Five patients had *PIK3CA* mutation: 1 in the acupuncture group (mPFS, 18 months) and 4 in the control group, with PFS of 1.27, 5.27, 10.50, and 20.97 months, respectively. Consistent with previous studies [22], higher level of PD-L1 expression was associated with a more favorable prognosis (Figure S9).

Two hundred and seven patients used PD-1 inhibitors, and the mPFS was 8.97 months and the mOS was 21.6 months. Patients who combined with chemotherapy and targeted therapy showed better mPFS (chemotherapy: 9.33 vs. 6.90 months; targeted therapy: 10.83 vs. 7.97 months; Figure S10-S11). Among the patients who received chemotherapy, survival was better with double-drug chemotherapy compared to either no chemotherapy or single-agent chemotherapy (double-drug chemotherapy *vs.* single-agent chemotherapy *vs.* no chemotherapy: mPFS, 9.90 vs. 6.37 vs. 6.87 months; mOS, 25.1 vs. 15.3 vs. 18.9 months, Figure S12). Patients treated with anlotinib had longer PFS (anlotinib vs. bevacizumab vs no targeted therapy: 13.17 vs. 9.93 vs. 7.97 months) and OS (23.6 vs. 20.5 vs. 21.6 months, Figure S13).

### Association of acupuncture with clinical outcomes

**Fig. 1:**
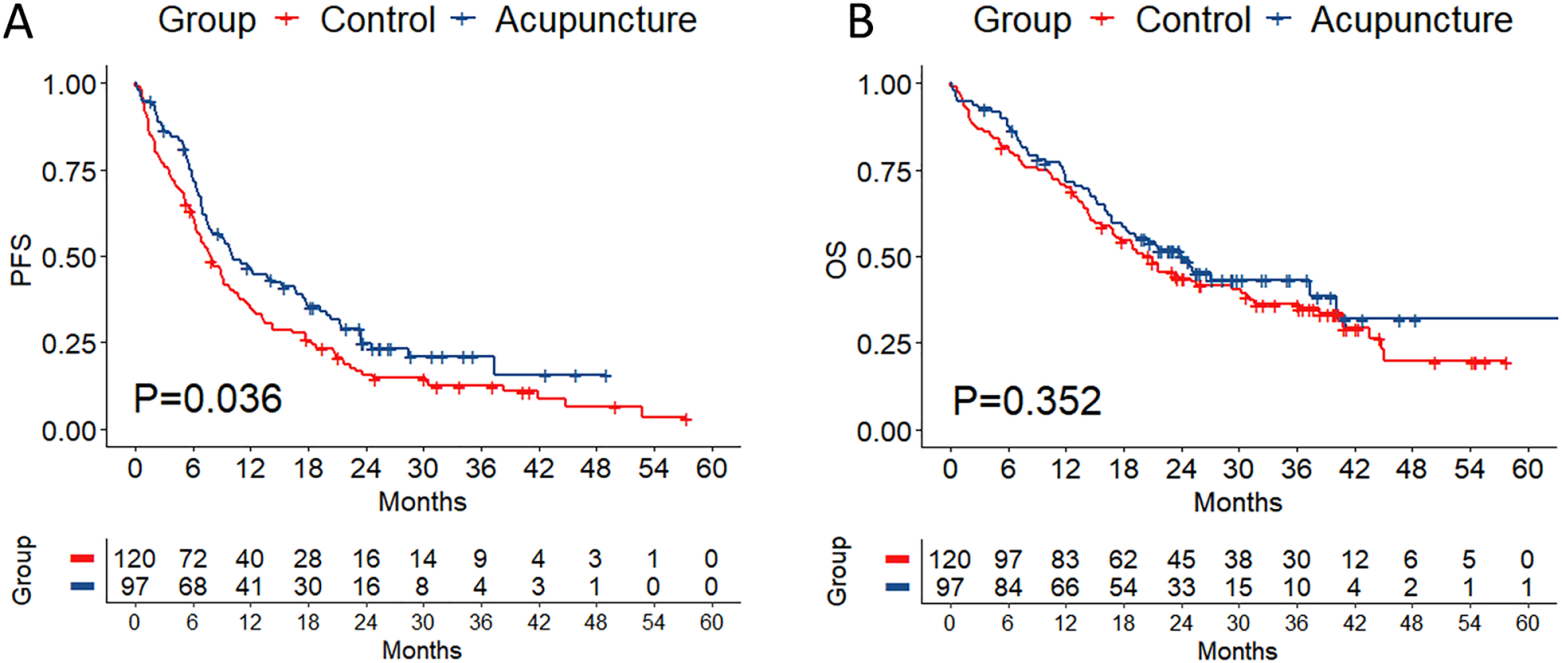
Overall survival curve of NSCLC patients stratified by acupuncture treatment

Compared to 120 patients in the control group, 97 patients in the acupuncture group presented better mPFS (10.23 vs. 7.87 months, *P* = 0.036, Fig. 1A). In terms of OS, patients in the acupuncture group showed an improvement, though it did not reach statistical significance (mOS, 24.1 vs. 20.9 months, *P* = 0.352, Fig. 1B).

### Multivariate Cox regression analysis

We performed Cox regression analysis using two data sets: the first was the original data, where missing values were directly removed; the second set included five datasets after multiple imputation and PSM matching (Table 2). Due to a high proportion of missing values (> 35%), PD-L1 expression and genetic status were excluded from the regression analysis.

**Table 2:** Cox regression analysis of progression-free survival and overall survival of the original population and propensity score matching population

Clinical characteristics		Original population				PSM population			
		HR 95% CI	*P*	aHR 95% CI	*P*	HR 95% CI	*P*	aHR 95% CI	*P*
Acupuncture(yes/no)	PFS	0.72 (0.54,0.98)	0.037	0.68 (0.44,1.05)	0.079	0.74 (0.54, 1.03)	0.074	0.65 (0.46, 0.91)	0.014
	OS	0.85 (0.60, 1.20)	0.352			0.82 (0.56, 1.19)	0.302		
Age	PFS	1.01 (0.99,1.03)	0.319			1.01 (0.99, 1.03)	0.318		
	OS	1.02 (1.00, 1.04)	0.074	1.01 (0.98,1.03)	0.695	1.02 (1.00, 1.04)	0.128	1.01 (0.99, 1.04)	0.311
Gender (Female/Male)	PFS	0.81 (0.54,1.22)	0.313			0.75 (0.46, 1.22)	0.243		
	OS	0.69 (0.42, 1.10)	0.120	1.35 (0.66,2.79)	0.414	0.69 (0.37, 1.28)	0.253		
Smoking History(yes/no)	PFS	1.02 (0.72,1.45)	0.919			1.03 (0.7, 1.52)	0.865		
	OS	1.18 (0.78, 1.77)	0.435			1.14 (0.71, 1.83)	0.583		
PS Score(> 2/≤ 2)	PFS	1.39 (1.07,1.82)	0.015	1.15 (0.81,1.63)	0.449	1.31 (0.99, 1.75)	0.062	1.26 (0.89, 1.78)	0.210
	OS	1.83 (1.37, 2.46)	< 0.001	1.59 (1.10,2.30)	0.014	1.72 (1.27, 2.34)	0.001	1.54 (1.09, 2.19)	0.018
Clinical Stage(IV/III)	PFS	1.48 (1.15,1.91)	0.003	9.80 (2.04,47.14)	0.004	1.44 (1.08, 1.92)	0.018	1.43 (0.95, 2.15)	0.094
	OS	1.67 (1.23, 2.28)	0.001	16.12 (3.01,86.17)	0.001	1.67 (1.14, 2.42)	0.013	1.69 (1.06, 2.69)	0.030
Pathology (others as ref.)									
Adenocarcinoma	PFS	1.22 (0.72,2.08)	0.464			1.38 (0.67, 2.82)	0.385		
	OS	1.46 (0.78, 2.76)	0.240	1.56 (0.64,3.79)	0.328	1.45 (0.62, 3.41)	0.396		
Squamous cell carcinoma	PFS	1.44 (0.82,2.51)	0.206			1.53 (0.75, 3.13)	0.248		
	OS	1.71 (0.88, 3.33)	0.113	3.83 (1.50,9.81)	0.005	1.53 (0.65, 3.57)	0.333		
The number of metastatic organ (0 as ref.)									
1	PFS	1.80 (0.90, 3.60)	0.095	0.10 (0.01,0.82)	0.032	1.55 (0.65, 3.67)	0.340	1.05 (0.36, 3.08)	0.934
	OS	1.65 (0.72, 3.81)	0.240	0.03 (0.00,0.31)	0.003	1.44 (0.45, 4.61)	0.559	0.75 (0.19, 3.04)	0.698
2	PFS	2.02 (0.97, 4.20)	0.059	0.07 (0.01,0.58)	0.013	1.66 (0.67, 4.09)	0.290	0.90 (0.27, 2.99)	0.867
	OS	3.07 (1.31, 7.2)	0.010	0.04 (0.00,0.39)	0.005	2.61 (0.79, 8.57)	0.148	0.96 (0.21, 4.29)	0.954
3	PFS	4.10 (1.93, 8.70)	< 0.001	0.13 (0.01,1.09)	0.060	3.19 (1.17, 8.71)	0.045	1.68 (0.50, 5.68)	0.420
	OS	4.95 (2.06, 11.91)	< 0.001	0.08 (0.01,0.76)	0.028	4.02 (1.17, 13.85)	0.053	1.51 (0.34, 6.66)	0.594
4	PFS	3.16 (1.38, 7.23)	0.006	0.07 (0.01,0.70)	0.024	2.33 (0.78, 6.96)	0.153	1.21 (0.32, 4.65)	0.782
	OS	3.28 (1.27,8.46)	0.014	0.04 (0.00,0.41)	0.007	2.84 (0.68, 11.88)	0.187	1.11 (0.2, 6.31)	0.907
≥ 5	PFS	5.16 (2.18, 12.21)	< 0.001	0.11 (0.01,1.06)	0.056	2.27 (0.76, 6.75)	0.172	1.69 (0.50, 5.73)	0.422
	OS	7.26 (2.74,19.27)	< 0.001	0.09 (0.01,0.90)	0.040	3.28 (0.76, 14.15)	0.155	1.99 (0.39, 10.27)	0.436
Liver metastasis (yes/no)	PFS	2.39 (1.48,3.85)	< 0.001	1.71 (0.80,3.66)	0.170	1.14 (0.75, 1.75)	0.540		
	OS	2.04 (1.24, 3.38)	0.005	1.30 (0.64,2.60)	0.468	1.08 (0.62, 1.88)	0.791		
Bone metastasis (yes/no)	PFS	1.72 (1.2,2.46)	0.003	1.55 (0.89,2.70)	0.119	1.54 (1.09, 2.17)	0.015	1.32 (0.81, 2.14)	0.266
	OS	2.36 (1.55, 3.58)	< 0.001	2.04 (1.12,3.72)	0.020	2.11 (1.42, 3.15)	< 0.001	1.35 (0.78, 2.33)	0.292
Brain metastasis (yes/no)	PFS	1.33 (0.87,2.02)	0.187	1.36 (0.80,2.30)	0.260	0.99 (0.68, 1.44)	0.942		
	OS	1.32 (0.83, 2.10)	0.243			0.94 (0.54, 1.63)	0.831		
The types of (other as ref.)									
PD-1 inhibitor	PFS	1.53 (0.62,3.78)	0.358			1.30 (0.17, 10.0)	0.801		
	OS	0.92 (0.38, 2.25)	0.857			0.92 (0.37, 2.26)	0.857		
PD-L1 inhibitor	PFS	1.69 (0.32,8.81)	0.535			1.51 (0.61, 3.74)	0.377		
	OS	1.23 (0.24, 6.35)	0.807			1.09 (0.16, 7.20)	0.930		
Chemotherapy(yes/no)	PFS	0.82 (0.58,1.16)	0.257			0.91 (0.59, 1.39)	0.649		
	OS	0.86 (0.57, 1.27)	0.442			1.01 (0.62, 1.65)	0.957		
Target therapy(yes/no)	PFS	0.88 (0.64,1.2)	0.401			0.85 (0.61, 1.19)	0.349		
	OS	1.01 (0.70, 1.45)	0.966			0.93 (0.63, 1.38)	0.727		

*PSM* propensity score matching, *HR* hazard ratios, *ECOG PS* Eastern cooperative oncology group performance status, *PD-L1* programmed cell death 1 ligand 1

In the original dataset, univariable Cox analysis identified number of metastatic organs, liver metastasis, bone metastasis, brain metastasis, PS score, clinical stage, and acupuncture as factors associated with PFS (*P* < 0.20). After adjusting for confounding factors in the multivariate Cox regression model, acupuncture was associated with a 32% reduction in the risk of disease progression (HR = 0.68, 95% CI 0.44–1.05, *P* = 0.079, N = 130, 87 samples were deleted due to incomplete information). In the PSM population (N = 194), number of metastatic organs, bone metastasis, clinical stage, acupuncture, and PS score were included in the multivariate model (*P* < 0.20), which ultimately showed that acupuncture was an independent predictor of PFS (*P* < 0.05).

In the univariate analysis for OS: In the original dataset, acupuncture was associated with a non-significant 15% reduction in mortality risk (HR = 0.85, 95% CI 0.60–1.20, *P* = 0.352). In the PSM population, the effect size slightly improved but remained non-significant, with an 18% risk reduction (HR = 0.82, 95% CI 0.56–1.19, *P* = 0.302).

### Effectiveness of acupuncture across subgroups

The subgroup analyses revealed differential effects of acupuncture across clinical populations (Table 3). Improved PFS was generally observed, though statistical significance varied. Notably, in the PD-L1-positive population, no significant survival difference was detected between acupuncture and control groups (PFS: *P* > 0.1; OS: *P* > 0.2). Conversely, among patients with bone metastasis, the patients in the acupuncture group demonstrated pronounced clinical benefits, with significantly prolonged PFS (*P* < 0.001) and OS (*P* = 0.049) compared to controls. For the association of acupuncture with OS, most subgroups exhibited non-significant trends favoring acupuncture (HR < 1, *P* > 0.05).

**Table 3:** The association of acupuncture with progression-free disease and overall survival across subgroups

Subgroups	Acupuncture group	Control group	PFS		OS	
			HR, 95% CI	P value	HR, 95% CI	P value
Smoking history						
No	20	32	0.53 (0.27,10.6)	0.073	0.68 (0.30,1.55)	0.359
Yes	76	87	0.79 (0.56,1.12)	0.189	0.88 (0.59,1.32)	0.545
Missing	1	1	–	–	–	–
ECOG PS						
< 2	76	77	0.83 (0.58,1.19)	0.312	0.96 (0.62,1.50)	0.872
≥ 2	18	22	0.73 (0.37,1.44)	0.359	0.70 (0.34,1.46)	0.345
Missing	3	21	0.36 (0.08,1.57)	0.173	0.91 (0.21,4.03)	0.905
Pathology						
Adenocarcinoma	62	68	0.62 (0.42,0.92)	0.018	0.84 (0.54,1.32)	0.452
Squamous cell carcinoma	29	39	0.88 (0.51,1.52)	0.650	0.83 (0.44,1.55)	0.554
Others	6	13	0.99 (0.34,2.90)	0.982	0.79 (0.20,3.07)	0.734
PD-L1						
< 1%	6	17	0.42 (0.14,1.31)	0.135	0.48 (0.14,1.70)	0.254
1–49%	24	24	1.33 (0.72,2.46)	0.366	1.27 (0.61,2.62)	0.520
≥ 50%	22	30	0.89 (0.45,1.76)	0.743	1.38 (0.62,3.06)	0.432
Missing	45	49	0.56 (0.35,0.88)	0.012	0.70 (0.41,1.19)	0.189
EGFR mutation						
No mutation	61	76	0.74 (0.51,1.08)	0.114	0.89 (0.57,1.38)	0.600
Mutation	0	4	–	–	–	–
Missing	36	40	0.71 (0.42,1.19)	0.197	0.69 (0.38,1.26)	0.225
Clinical stage						
IIIA	10	6	0.21 (0.05,0.85)	0.029	0.52 (0.07,3.72)	0.516
IIIB	14	16	1.19 (0.50,2.84)	0.695	1.18 (0.42,3.30)	0.750
IIIC	5	7	5.21 (1.2,22.57)	0.028	1.33 (0.30,5.99)	0.707
IV	67	79	0.64 (0.45,0.92)	0.016	0.83 (0.55,1.24)	0.356
Missing	1	12	5.48 (0.5,60.52)	0.165	–	–
The number of metastatic organ						
0	6	10	1.02 (0.28,3.74)	0.971	1.32 (0.29,5.91)	0.717
1	29	30	0.83 (0.46,1.51)	0.547	0.89 (0.42,1.89)	0.764
2	18	17	0.59 (0.27,1.25)	0.168	0.73 (0.32,1.62)	0.436
3	9	15	0.54 (0.23,1.27)	0.157	0.78 (0.31,1.99)	0.605
4	6	9	0.33 (0.10,1.12)	0.075	1.47 (0.42,5.10)	0.543
≥ 5	5	7	0.89 (0.27,3.00)	0.855	0.89 (0.27,3.00)	0.855
Missing	24	32	0.70 (0.37,1.33)	0.278	0.70 (0.31,1.58)	0.392
Liver metastasis						
No	65	66	0.78 (0.53,1.14)	0.199	0.90 (0.58,1.40)	0.647
Yes	7	17	0.85 (0.33,2.23)	0.746	1.68 (0.61,4.65)	0.314
Missing	25	37	0.69 (0.37,1.27)	0.232	0.62 (0.28,1.34)	0.223
Bone metastasis						
No	33	42	1.27 (0.75,2.13)	0.376	1.28 (0.67,2.43)	0.455
Yes	36	38	0.31 (0.18,0.52)	0.000	0.59 (0.34,1.00)	0.049
Missing	28	40	0.70 (0.39,1.26)	0.236	0.76 (0.38,1.52)	0.436
Brain metastasis						
No	55	60	0.62 (0.41,0.93)	0.022	0.77 (0.48,1.25)	0.290
Yes	13	20	1.20 (0.56,2.60)	0.637	1.11 (0.47,2.63)	0.813
Missing	29	40	0.81 (0.46,1.43)	0.475	0.96 (0.49,1.86)	0.899
The type of ICI						
PD-1	93	114	0.71 (0.52,0.97)	0.031	0.82 (0.57,1.18)	0.279
PD-L1	1	2	–	–	–	–
Other	3	4	1.70 (0.28,10.49)	0.566	–	–
Chemotherapy						
No	16	34	0.66 (0.33,1.32)	0.240	0.69 (0.31,1.55)	0.371
Yes	81	86	0.77 (0.55,1.08)	0.132	0.92 (0.61,1.37)	0.668
Targeted therapy						
No	62	83	0.70 (0.48,1.02)	0.066	0.85 (0.55,1.31)	0.449
Yes	35	37	0.77 (0.46,1.28)	0.311	0.85 (0.46,1.56)	0.599

*HR* hazard ratios, *ECOG PS* Eastern cooperative oncology group performance status, *PD-L1* programmed cell death 1 ligand 1, *EGFR* epidermal growth factor receptor

To further explore effect modification, interaction models were incorporated into Cox regression analyses, evaluating interactions between acupuncture and key clinical features: smoking history, ECOG PS, histology, clinical stage, PD-L1 expression level, number of metastatic organs, bone/liver/brain metastasis, and combination with chemotherapy/targeted therapy. Among 149 patients with available bone metastasis data, bone metastasis was strongly associated with worse prognosis (PFS: HR = 3.87, *P* < 0.001; OS: HR = 3.33, *P* < 0.001). However, the combination with acupuncture significantly attenuated this risk through a protective interaction (PFS: HR = 0.24, *P* < 0.001; OS: HR = 0.47, *P* = 0.075). In contrast, among 123 patients with PD-L1 data, positive PD-L1 expression predicted better survival, but acupuncture appeared to partially diminish this protective effect (interaction HR > 2.0). No significant interactions were observed for other clinical features (Table 4).

**Table 4:** Interaction of acupuncture with bone metastasis and PD-L1 expression

	PFS			OS		
	HR	SE	*P* value	HR	SE	*P* value
Bone metastasis (no/yes, N = 149)						
Acupuncture	1.25	0.265	0.392	1.24	0.326	0.505
Bone metastasis	3.87	0.259	< 0.001	3.33	0.282	< 0.001
Acupuncture*Bone metastasis	0.24	0.373	< 0.001	0.47	0.424	0.075
PD-L1 expression (< 1%/1–49%/≥ 50%, N = 123)						
Acupuncture	0.39	0.562	0.094	0.50	0.643	0.285
PD-L1 1–49%	0.43	0.339	0.013	0.42	0.379	0.021
PD-L1 ≥ 50%	0.36	0.327	0.002	0.34	0.369	0.003
Acupuncture*PD-L1 1–49%	3.44	0.647	0.055	2.59	0.737	0.197
Acupuncture*PD-L1 ≥ 50%	2.15	0.656	0.243	2.46	0.748	0.228

*PD-L1* programmed cell death 1 ligand 1

## Discussion

This study represents the first investigation into the effectiveness of acupuncture on ICIs-treated patients with NSCLC. Our findings suggest that acupuncture in combination with ICIs holds potential to enhance clinical outcomes, particularly in patients with bone metastasis. However, in patients with positive PD-L1 expression, acupuncture did not show a trend toward improvement.

The acupuncture group demonstrated pronounced survival benefits in the subgroup of NSCLC patients with bone metastases, evidenced by significantly prolonged PFS (*P* < 0.001) and OS (*P* = 0.049). Bone metastases occur in 30–40% of NSCLC cases [23], with most patients experiencing cancer-related pain. In clinical practice, acupuncture was commonly used for symptom management, rather than survival improvement. However, established evidence indicates that effective symptom control enhances physical status, treatment tolerance, and quality of life, thereby indirectly contributing to extended survival [23, 24]. This analgesic-mediated improvement in symptom burden may partially explain the benefit observed in this bone metastasis subgroup.

In addition to pain relief, the immunomodulatory effect of acupuncture may play an important role in the survival benefit. Bone metastases drive systemic immunosuppression through a self-perpetuating"vicious cycle"[25, 26]: Tumor cells in bone secrete cytokines (IL-1, IL-6, and TNF-α) that activate osteoclasts, disrupting bone homeostasis and releasing pro-tumor factors (TGF-β, and IGF) that further fuel tumor growth and reshape the microenvironment. This process triggers immunosuppressive cascades, including suppression of Th1 effector T cells and loss of type I interferon signaling, which cripples dendritic cell maturation and cytotoxic immune cell function [27]. Consistent with these findings, our previous study has found that patients with bone metastases exhibited a'cold'TIME [28], characterized by a concomitant reduction in effector immune markers (CD3^+^, CD4^+^, CD4^+^, and CD68^+^) and diminished expression of inhibitory checkpoints (PD-L1, TIM-3, and LAG-3)—a dual deficiency reflecting systemic immune inertness that cripples both tumoricidal activity and ICI-driven immunomodulatory potential. Interestingly, acupuncture has demonstrated immunomodulatory properties capable of reshaping the TIME [18, 19]. These findings suggest that acupuncture may enhance the effectiveness of ICIs by transforming immunologically"cold"tumors into"hot"phenotypes. Specifically, patients with bone metastasis, who predominantly exhibited"cold"tumors characterized by the absence of cytotoxic lymphocyte infiltration and inhibitory checkpoint expression [28]. Furthermore, the current study indicated acupuncture in combination with ICIs was associated with significant clinical improvements in NSCLC patients with bone metastasis. Taken together, the results suggest that NSCLC patients with bone metastasis are responsive to acupuncture, likely benefiting from TIME remodeling.

PD-L1 was a well-established predictor of ICI responsiveness, where higher level was generally associated with improved clinical outcomes in ICI-treated NSCLC patients [22, 29]. However, acupuncture did not show the similar effectiveness in this subgroup, and instead, there was even a trend towards poor effectiveness. Intriguingly, this finding reinforces the hypothesized mechanism of acupuncture—facilitating"cold-to-hot"tumor conversion. In active TIME, increased cytotoxic activity triggers negative feedback mechanisms that upregulate inhibitory checkpoints, such as PD-L1 and LAG-3, creating a hallmark"hot tumor"signature of concurrent high-level of cytotoxic and immunosuppressive markers [30]. Thus, a high level of PD-L1 expression generally signify a pre-existing"hot"state where immune activity is already existed. In such contexts, acupuncture may not produce the comparable activation of TIME as patients with bone metastasis.

## Limitation

This study has several limitations: (1) The retrospective nature of this study is a primary limitation, which precludes causal inferences, though propensity score matching mitigated selection bias; (2) Heterogeneous acupuncture protocols (frequency, duration and acupoint selection) may have diluted treatment effects; (3) Sparse PD-L1 and genomic data limited adjustment for key confounders. Well-designed, prospective randomized controlled studies are needed to verify the efficacy of acupuncture.

## Conclusion

Acupuncture was associated with improved survival for ICI-treated NSCLC, yet there is heterogeneity among various subgroups. Specifically, it exhibits significant survival benefits in patients with bone metastases, whereas in cases of high PD-L1 expression, it showed no trend of improvement and may even have potential adverse effects. For patients with negative factors associated with immunotherapy, incorporating acupuncture into oncology care could be a better treatment option.

## Supplementary Information

Supplementary material 1. Overall survival curve of NSCLC patients grouped by different clinical characteristics.

Supplementary material 2. Interaction of acupuncture with other characteristics.

## Data Availability

The datasets and materials supporting the conclusions of this study are available from the corresponding author upon reasonable request. Interested researchers may contact Hai-bo zhang to request access to the data. Data sharing will comply with institutional ethical guidelines.

## Abbreviations

CIs: Confidence intervals
EGFR: Epidermal growth factor receptor
ECOG PS: Eastern cooperative oncology group performance status
HRs: Hazard ratios
ICIs: Immune checkpoint inhibitors
NSCLC: Non-small cell lung cancer
OS: Overall survival
PFS: Progression-free survival
PSM: Propensity score matching
RECIST: Response evaluation criteria in solid tumors
TIME: Tumor immune microenvironment
TME: Tumor microenvironment
