# Supplementary material for: The association between acupuncture and response to immune checkpoint inhibitors in non-small cell lung cancer
Source: Chin Med. 2025 Sep 19;20:145. doi: 10.1186/s13020-025-01148-4 (PMC12447595; doi:10.1186/s13020-025-01148-4)

Supplementary Figure 1A, PFS of NSCLC patients stratified by smoking history.; 2B, OS of NSCLC patients stratified by smoking history.

A B


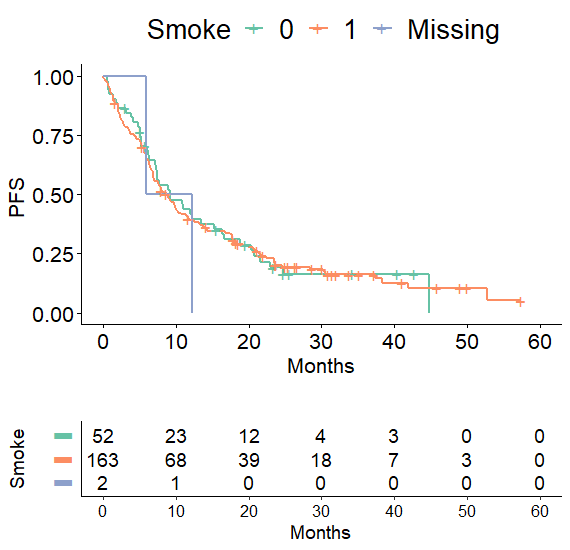

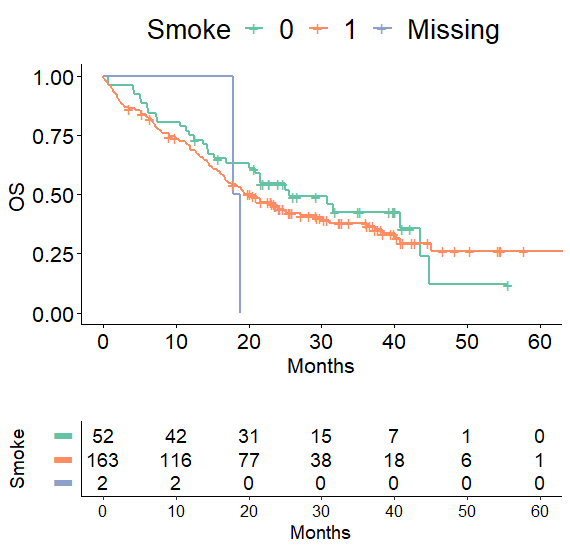


Supplementary Figure 2A, PFS of NSCLC patients stratified by pathology; 2B, OS of NSCLC patients stratified by pathology.

A B


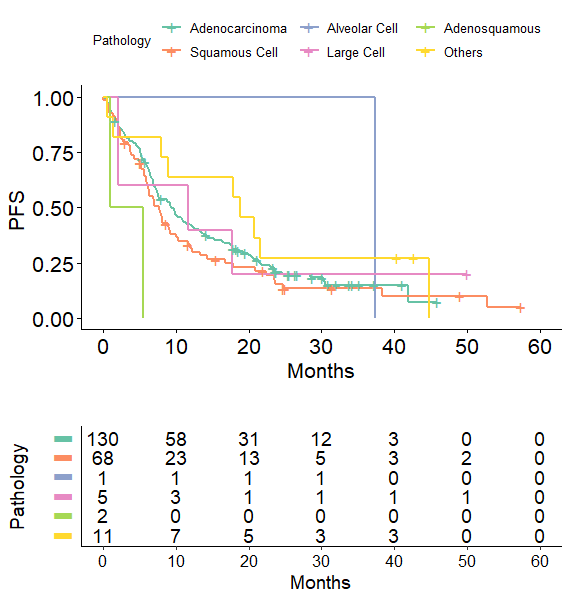

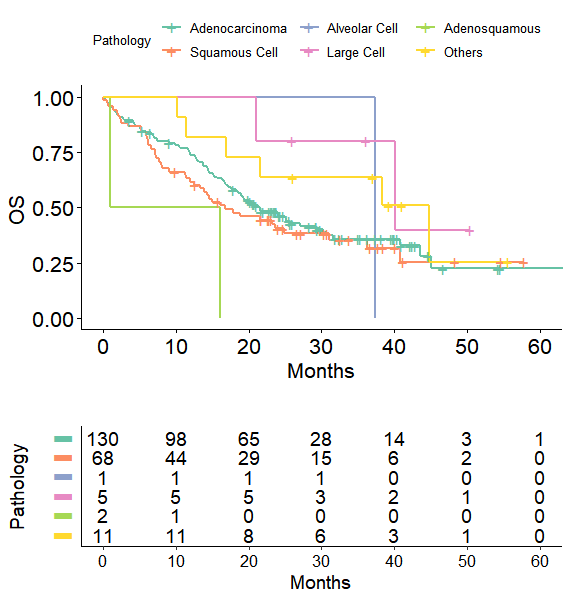


Supplementary Figure 3A, PFS of NSCLC patients stratified by ECOG-PS; 3B, OS of NSCLC patients stratified by ECOG-PS.

A B


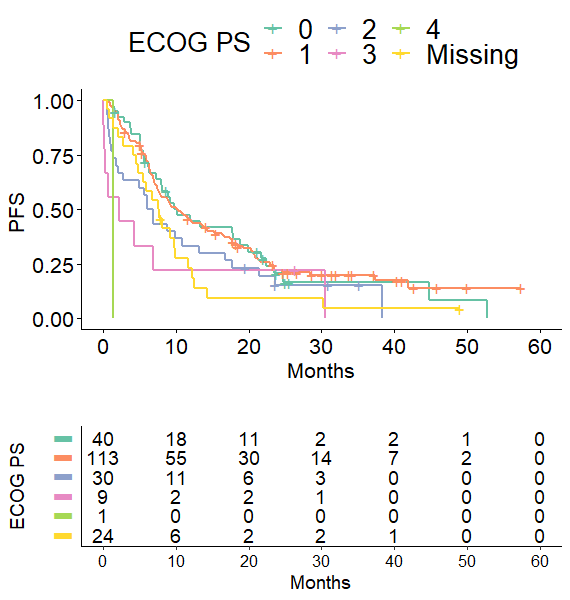

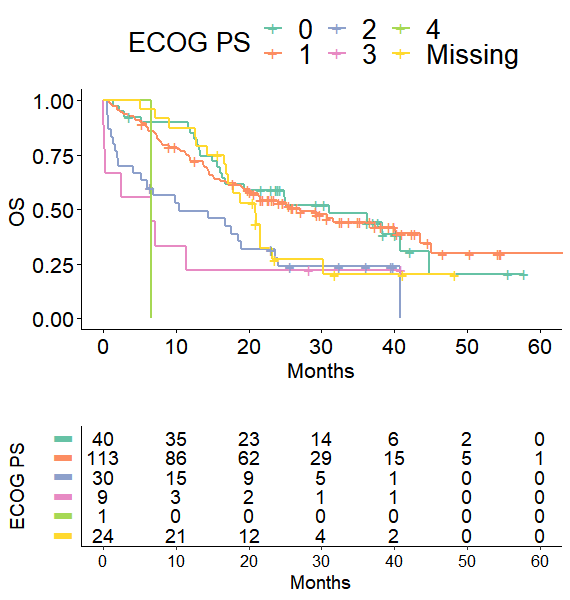


Supplementary Figure 4A, PFS of NSCLC patients stratified by clinical stage; 4B, OS of NSCLC patients stratified by clinical stage.

A B


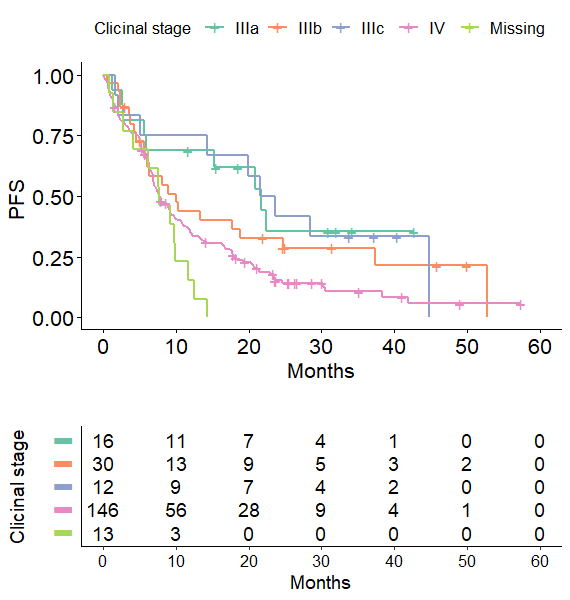

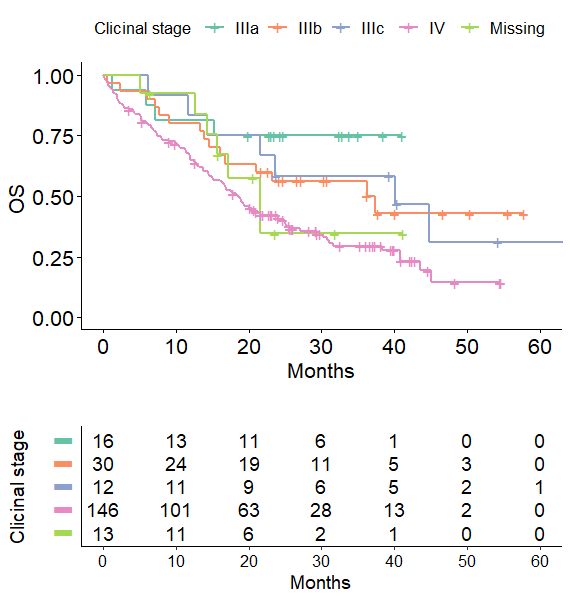


Supplementary Figure 5A, PFS of NSCLC patients stratified by the number of metastatic organ; 5B, OS of NSCLC patients stratified by the number of metastatic organ.

A B


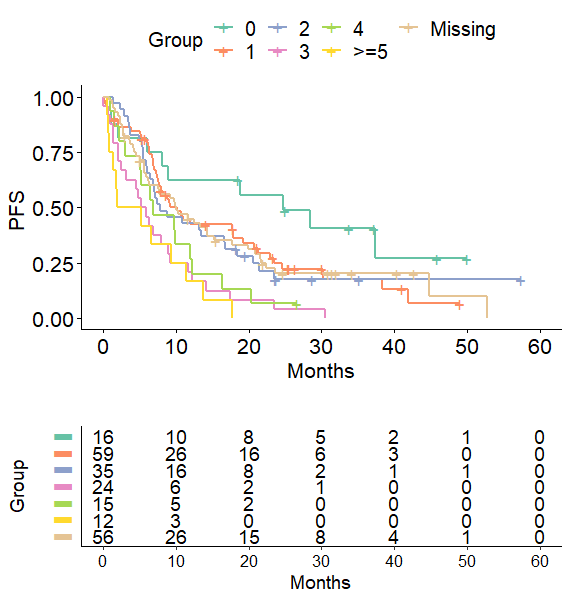

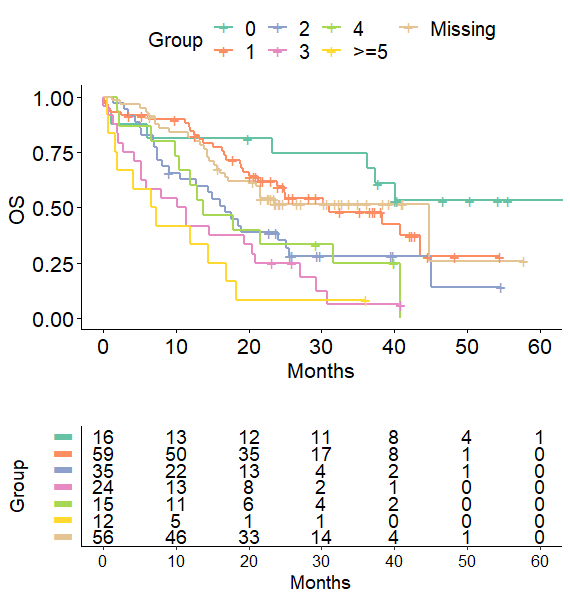


Supplementary Figure 6A, PFS of NSCLC patients stratified by liver metastasis; 6B, OS of NSCLC patients stratified by liver metastasis.

A B


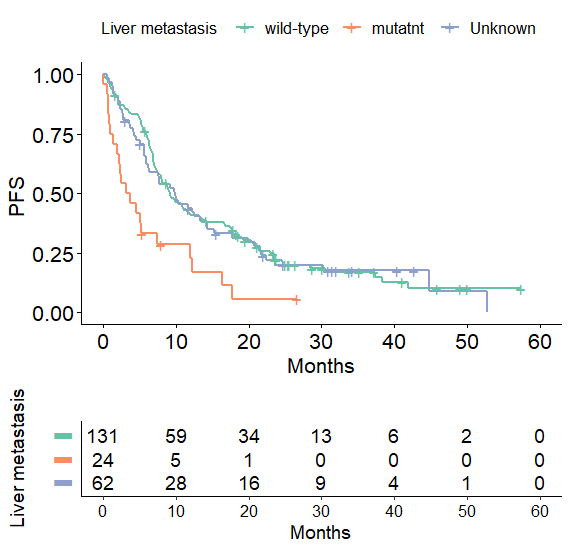

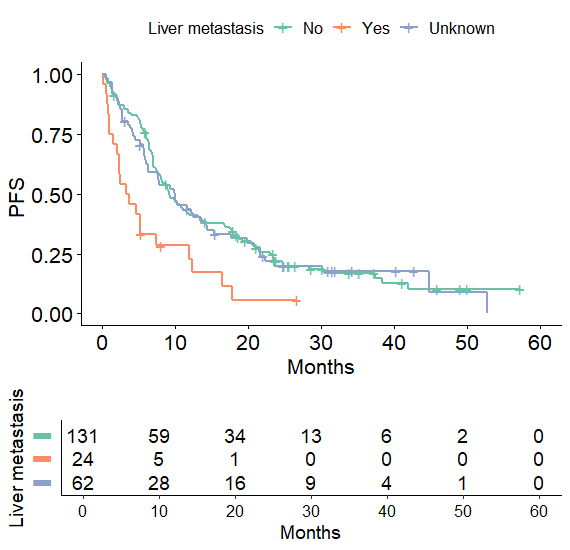


Supplementary Figure 7A, PFS of NSCLC patients stratified by bone metastasis; 7B, OS of NSCLC patients stratified by bone metastasis.

A B


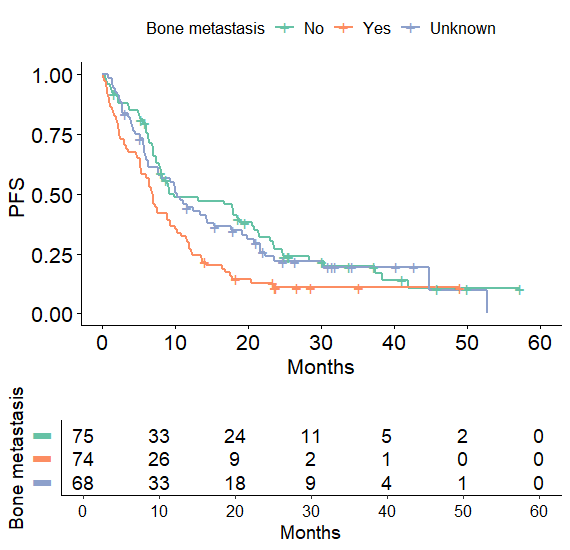

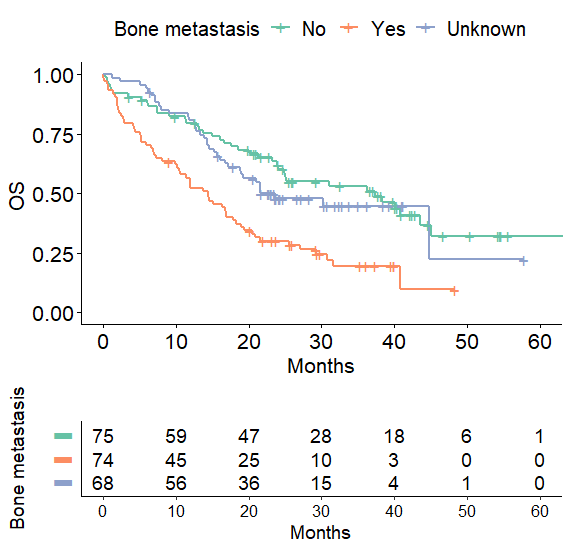


Supplementary Figure 8A, PFS of NSCLC patients stratified by brain metastasis; 8B, OS of NSCLC patients stratified by brain metastasis.

A B


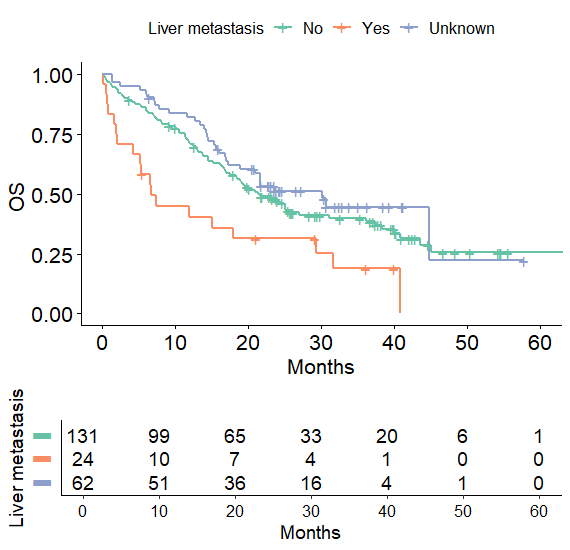

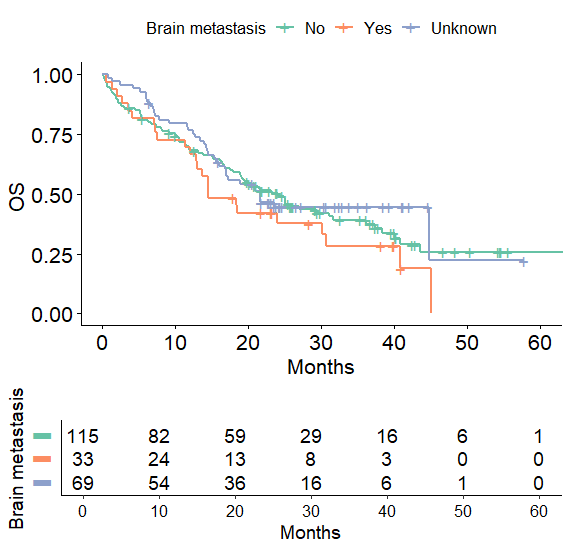


Supplementary Figure 9A, PFS of NSCLC patients stratified by PD-L1 expression; 9B, OS of NSCLC patients stratified by PD-L1 expression.

A B


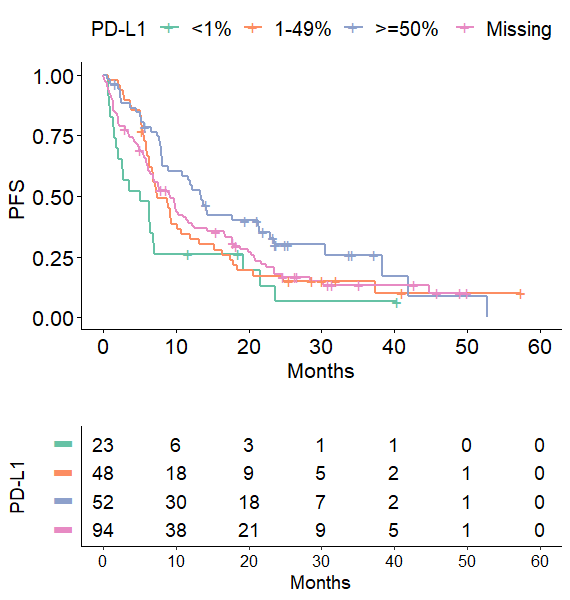

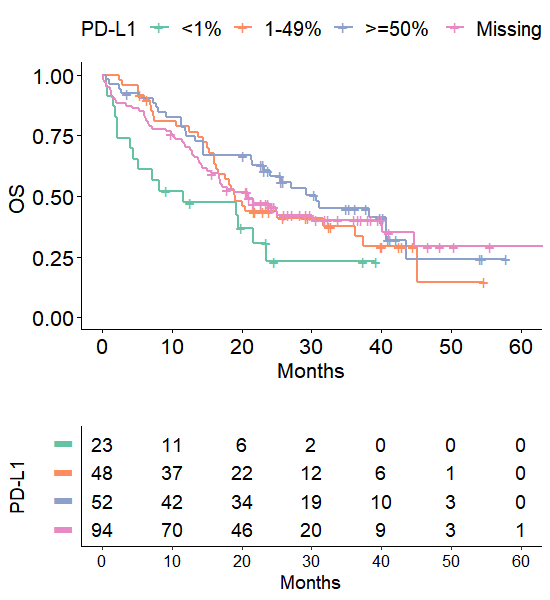


Supplementary Figure 10A, PFS of NSCLC patients stratified by chemotherapy use; 10B, OS of NSCLC patients stratified by chemotherapy use.

A B


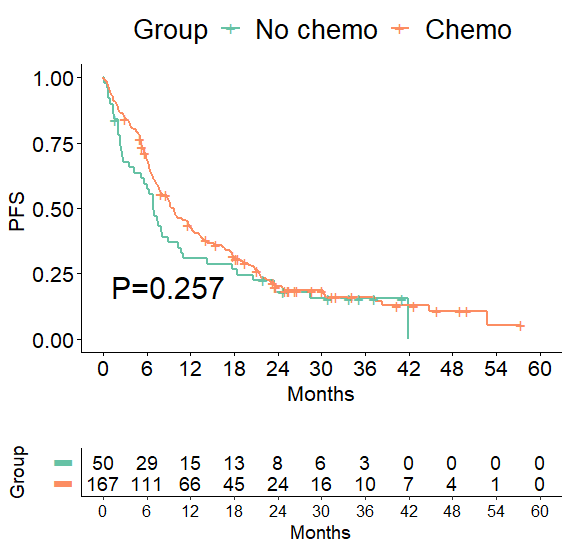

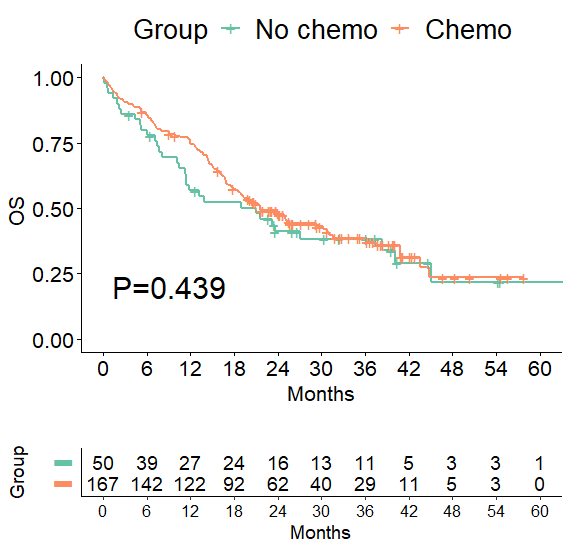


Supplementary Figure 11A, PFS of NSCLC patients grouped by targeted therapy use; 11B, OS of NSCLC patients grouped by targeted therapy use.

A B


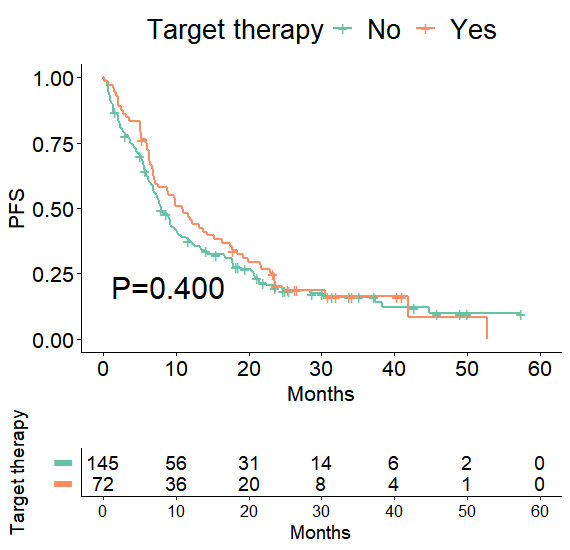

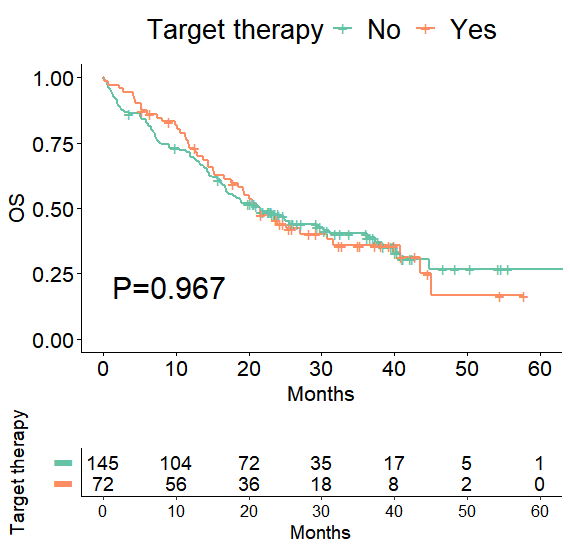


Supplementary Figure 12A, PFS of NSCLC patients grouped by chemotherapy regimens; 11B, OS of NSCLC patients grouped by chemotherapy regimens.

A B


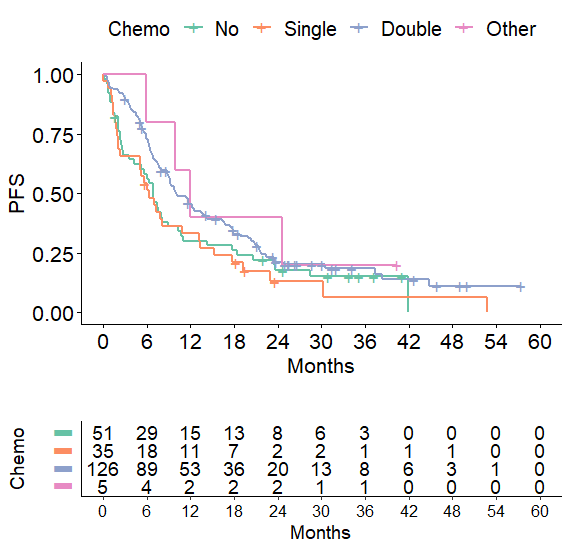

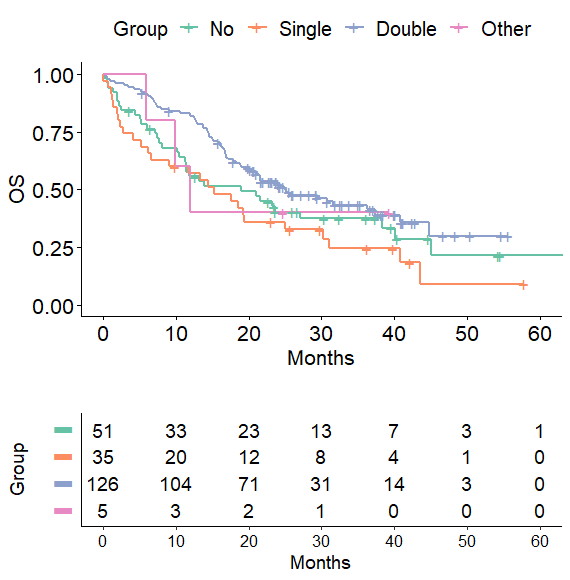


Supplementary Figure 13A, PFS of NSCLC patients grouped by targeted therapy regimens; 11B, OS of NSCLC patients grouped by targeted therapy regimens.

A B


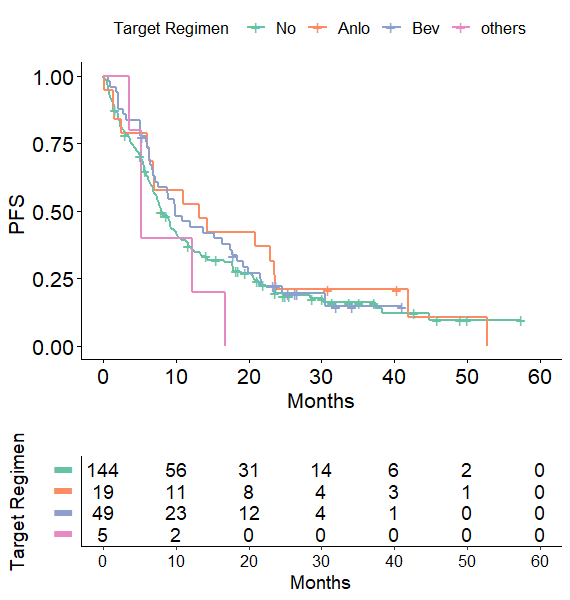

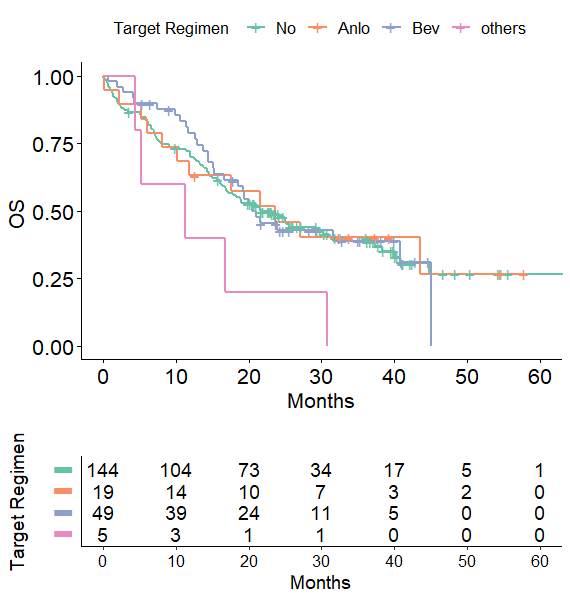

Supplement: Supplementary file 1 — Supplementary material 1. Overall survival curve of NSCLC patients grouped by different clinical characteristics. [file 13020_2025_1148_MOESM1_ESM.docx]
