# Supplementary material for: The association between acupuncture and response to immune checkpoint inhibitors in non-small cell lung cancer
Source: Chin Med. 2025 Sep 19;20:145. doi: 10.1186/s13020-025-01148-4 (PMC12447595; doi:10.1186/s13020-025-01148-4)
Supplement: Supplementary file 2 — Supplementary material 2. Interaction of acupuncture with other characteristics. [file 13020_2025_1148_MOESM2_ESM.docx]

Smoking history (no /yes)

| N=215 | PFS | | | OS | | |
| --- | --- | --- | --- | --- | --- | --- |
|  | HR | SE | *P* value | HR | SE | *P* value |
| Acupuncture | 0.53 | 0.344 | 0.068 | 0.63 | 0.414 | 0.264 |
| Smoke | 0.91 | 0.219 | 0.652 | 1.07 | 0.251 | 0.790 |
| Acupuncture*smoke | 1.48 | 0.386 | 0.309 | 1.42 | 0.460 | 0.447 |

ECOG PS (>=2 /<2)

| N=193 | PFS | | | | OS | | |
| --- | --- | --- | --- | --- | --- | --- | --- |
|  | HR | SE | *P* value | HR | | SE | *P* value |
| Acupuncture | 0.84 | 0.185 | 0.358 | 0.95 | | 0.222 | 0.807 |
| ECOG PS >=2 | 1.80 | 0.253 | 0.020 | 2.72 | | 0.276 | <0.001 |
| Acupuncture*ECOG PS >=2 | 0.76 | 0.392 | 0.486 | 0.72 | | 0.430 | 0.439 |

Pathology (Other /Squamous cell carcinoma / Adenocarcinoma)

| N=217 | PFS | | | OS | | |
| --- | --- | --- | --- | --- | --- | --- |
|  | HR | SE | *P* value | HR | SE | *P* value |
| Acupuncture | 0.92 | 0.540 | 0.882 | 0.72 | 0.678 | 0.625 |
| Squamous cell carcinoma (SCC) | 1.46 | 0.347 | 0.276 | 1.64 | 0.404 | 0.220 |
| Adenocarcinoma | 1.50 | 0.332 | 0.220 | 1.42 | 0.385 | 0.367 |
| Acupuncture*SCC | 0.98 | 0.607 | 0.969 | 1.19 | 0.749 | 0.819 |
| Acupuncture*Adenocarcinoma | 0.68 | 0.576 | 0.498 | 1.18 | 0.716 | 0.815 |

Stage (III /IV)

| N=204 | PFS | | | OS | | |
| --- | --- | --- | --- | --- | --- | --- |
|  | HR | SE | *P* value | HR | SE | *P* value |
| Acupuncture | 0.95 | 0.321 | 0.874 | 0.99 | 0.394 | 0.985 |
| Stage IV | 2.10 | 0.243 | 0.002 | 2.24 | 0.299 | 0.007 |
| Acupuncture*Stage IV | 0.68 | 0.369 | 0.303 | 0.83 | 0.444 | 0.666 |

The number of metastatic organ

| N=161 | PFS | | | OS | | |
| --- | --- | --- | --- | --- | --- | --- |
|  | HR | SE | *P* value | HR | SE | *P* value |
| Acupuncture | 0.92 | 0.306 | 0.788 | 0.84 | 0.354 | 0.618 |
| The number of metastatic organ | 1.42 | 0.071 | <0.001 | 1.38 | 0.078 | <0.001 |
| Acupuncture*The number of metastatic organ | 0.87 | 0.113 | 0.231 | 1.04 | 0.121 | 0.743 |

Liver metastasis (no /yes)

| N=155 | PFS | | | OS | | |
| --- | --- | --- | --- | --- | --- | --- |
|  | HR | SE | *P* value | HR | SE | *P* value |
| Acupuncture | 0.78 | 0.196 | 0.215 | 0.91 | 0.225 | 0.691 |
| Liver metastasis | 2.46 | 0.298 | 0.003 | 1.71 | 0.318 | 0.090 |
| Acupuncture*Liver metastasis | 0.82 | 0.524 | 0.701 | 1.81 | 0.543 | 0.273 |

Brain metastasis (no /yes)

| N=148 | PFS | | | OS | | |
| --- | --- | --- | --- | --- | --- | --- |
|  | HR | SE | *P* value | HR | SE | *P* value |
| Acupuncture | 0.62 | 0.212 | 0.023 | 0.77 | 0.244 | 0.288 |
| Brain metastasis | 0.99 | 0.281 | 0.978 | 1.15 | 0.302 | 0.643 |
| Acupuncture*Brain metastasis | 1.85 | 0.434 | 0.156 | 1.37 | 0.487 | 0.518 |

Combination of chemotherapy (no /yes)

| N=217 | PFS | | | OS | | |
| --- | --- | --- | --- | --- | --- | --- |
|  | HR | SE | *P* value | HR | SE | *P* value |
| Acupuncture | 0.61 | 0.352 | 0.160 | 0.69 | 0.409 | 0.363 |
| Chemotherapy | 0.80 | 0.215 | 0.283 | 0.80 | 0.245 | 0.368 |
| Acupuncture*Chemotherapy | 1.27 | 0.393 | 0.548 | 1.32 | 0.456 | 0.548 |

Combination of chemotherapy (no /yes)

| N=217 | PFS | | | OS | | |
| --- | --- | --- | --- | --- | --- | --- |
|  | HR | SE | *P* value | HR | SE | *P* value |
| Acupuncture | 0.70 | 0.192 | 0.066 | 0.84 | 0.222 | 0.445 |
| Targeted therapy | 0.85 | 0.211 | 0.440 | 1.01 | 0.239 | 0.956 |
| Acupuncture*Targeted therapy | 1.11 | 0.323 | 0.752 | 1.00 | 0.375 | 0.991 |
